# A novel mechanism underlying pathogenesis of Down syndrome

**DOI:** 10.1186/1750-1326-8-S1-O23

**Published:** 2013-09-13

**Authors:** Xin Wang, Yingjun Zhao, Xiaofei Zhang, Ying Zhou, Barbara Ranscht, Fred H Gage, William C Mobley, Yun-wu Zhang, Stuart A Lipton, Wanjin Hong, Huaxi Xu

**Affiliations:** 1 Sanford-Burnham Medical Research Institute, La Jolla, California, USA; 2 Fujian Provincial Key Laboratory of Neurodegenerative Disease and Aging Research, Medical College, Xiamen University, Xiamen, China; 3 Laboratory of Genetics, The Salk Institute for Biological Studies, La Jolla, California, USA; 4 Department of Neurosciences, University of California San Diego, La Jolla, California, USA; 5 Institute of Molecular and Cell Biology, Singapore

## Background

Down syndrome (DS) patients unanimously develop pathologies of Alzheimer’s disease (AD) in their 30s or 40s. Since DS is caused by the existence of an extra copy of chromosome 21 in cells, an over-dosage of genes on chromosome 21 may play a role in the pathogenesis of DS and perhaps AD as well. This idea has been well-supported in the case of the *APP* gene over-dosage which leads to overproduction of the neurotoxic β-amyloid (Aβ) peptides, the prime culprit in AD pathogenesis. However, the involvement of other genes on chromosome 21 in AD has barely been studied. Sorting nexin 27 (SNX27), a brain-enriched PDZ domain protein, regulates endocytic sorting and trafficking.

## Methods and results

Recently, we showed [1] that *Snx27*^-/-^ mice have severe neuronal pathology in the hippocampus and cortex. Although *Snx27*^+^*^/-^* mice have grossly normal neuroanatomy, we found defects in synaptic function, learning and memory and a reduction in the amounts of ionotropic glutamate receptors (NMDA and AMPA receptors) in these mice. SNX27 interacts with these receptors through its PDZ domain, regulating their recycling to the plasma membrane. We demonstrated a concomitant reduced expression of SNX27 and CCAAT/enhancer binding protein β (C/EBPβ) in Down’s syndrome brains and identify C/EBPβ as a transcription factor for *SNX27.* Down’s syndrome causes overexpression of miR-155, a chromosome 21-encoded microRNA that negatively regulates C/EBPβ, thereby reducing SNX27 expression and resulting in synaptic dysfunction. Upregulating SNX27 in the hippocampus of Down’s syndrome mice rescues synaptic and cognitive deficits. In addition to its role in DS, we also found that over-expression of SNX27 dissociates PS1/γ-secretase complex and reduces the level/ activity of γ-secretase and the production of Aβ, whereas and depletion of SNX27 results in increased γ-secretase activity and Aβ production.

## Conclusion

Our identification of the role of SNX27 in synaptic function and in regulating γ-secretase activity and Aβ generation establishes a new molecular mechanism for pathogenesis of both DS and AD.
